# Attractiveness Modulates Neural Processing of Infant Faces Differently in Males and Females

**DOI:** 10.3389/fnhum.2017.00551

**Published:** 2017-11-14

**Authors:** Lijun Yin, Mingxia Fan, Lijia Lin, Delin Sun, Zhaoxin Wang

**Affiliations:** ^1^Shanghai Key Laboratory of Brain Functional Genomics (Ministry of Education), Institute of Cognitive Neuroscience, School of Psychology and Cognitive Science, East China Normal University, Shanghai, China; ^2^Department of Psychology, Sun Yat-sen University, Guangzhou, China; ^3^Shanghai Key Laboratory of MR, East China Normal University, Shanghai, China; ^4^Brain Imaging and Analysis Center, Duke University Medical Center, Durham, NC, United States

**Keywords:** infant, face, attractiveness, self, other, reward, gender difference

## Abstract

Consistent attention and proper processing of infant faces by adults are essential for infant survival. Previous behavioral studies showed gender differences in processing infant cues (e.g., crying, laughing or facial attractiveness) and more importantly, the efforts invested in nurturing offspring. The underlying neural mechanisms of processing unknown infant faces provide hints for understanding behavioral differences. This functional magnetic resonance imaging (fMRI) study recruited 32 unmarried adult (16 females and 16 males) participants to view unfamiliar infant faces and rate the attractiveness. Adult faces were also included. Behaviorally, despite that females and males showed no differences in attractiveness ratings of infant faces, a positive correlation was found between female’s (but not male’s) subjective liking for infants and attractiveness ratings of the infant faces. Functionally, brain activations to infant faces were modulated by attractiveness differently in males and females. Specifically, in female participants, activities in the ventromedial prefrontal cortex (vmPFC) and striatum/Nucleus Accumbens (NAcc) were positively modulated by infant facial attractiveness, and the modulation coefficients of these two regions were positively correlated. In male participants, infant facial attractiveness negatively modulated the activity in the dorsomedial prefrontal cortex (dmPFC). Our findings reveal that different neural mechanisms are involved in the processing of infant faces, which might lead to observed behavioral differences between males and females towards the baby.

## Introduction

Human babies are extremely vulnerable and completely dependent upon adults’ care in order to survive (Senese et al., 2013). Despite the fact that both males and females take care of their infants to a certain extent, an intriguing fact is that women typically spend 2–3 times more with babies than men (Rossi, 1984; Mitchell et al., 2005; Bault et al., 2011). Besides, an overwhelming 97.7%–99.6% of prekindergarten and kindergarten teachers are female (e.g., U.S. Educational Statistics Yearbook of China, 2010; U.S. Bureau of the Census, 2011). Also, gender differences in the motivational processing of babies have been found in many behavioral studies (Doucet, 2009; Yamamoto et al., 2009; Hahn et al., 2013). In a study about preference, females showed greater preference for infants than males, even at the age of 4 months (Maestripieri and Pelka, 2002). With regards to the neuroimaging studies, infant cues (e.g., crying, laughing, faces and etc.) have been found to elicit different neural patterns in males and females. Infant laughter and cries elicited activation in the amygdala and anterior cingulate of women, whereas the control stimuli elicited stronger activations in men (Sander et al., 2007). Independently of parental status, females but not males showed neural deactivation in the anterior cingulate cortex in response to both infant crying and laughing (Seifritz et al., 2003). In an EEG study, the baby-specific N1 response was much stronger in women than in men across the left hemisphere (Proverbio et al., 2011). When participants viewed faces of their own infants’, rather than the faces of unfamiliar infants and adults, stronger neural activation in several regions, including the orbitofrontal cortex and anterior insula, have been found (Nitschke et al., 2004; Ranote et al., 2004; Kringelbach et al., 2008; Noriuchi et al., 2008; Strathearn et al., 2008).

Central to parental care is adults’ ability to process infant cues (Parsons et al., 2014). One essential infant cue is facial attractiveness. An infant facial attractiveness or cuteness has been considered as an innate releasing mechanism for caretaking behaviors and affective orientation toward infants (Lorenz, 1943; Lobmaier et al., 2010; Hahn et al., 2013). Existing evidence suggests that there are consistent gender differences when it comes to processing infant attractiveness. Although both males and females show similar performance in detecting emotional valence and age (Parsons et al., 2011), compared to men, women were generally more perceptive and responsive to infant facial attractiveness (Parsons et al., 2011) and performed better in detecting attractive gradations of infant faces (Sprengelmeyer et al., 2009; Lobmaier et al., 2010). Moreover, infants who had relatively cuter faces elicited stronger caretaking motivation and higher activity in the striatum/nucleus accumbens (NAcc) in women (Glocker et al., 2009a,b). Thus understanding the underlying neural mechanisms of processing infant faces helps to understand established behavioral differences.

On one hand, the reward associated regions (e.g., the striatum/NAcc) might be the regions of interest in processing infant faces. On the other hand, self-resemblance, a putative cue of relatedness, might also play an important role in processing infant cues, especially in males. Both men and women are attracted to infant faces that look like their own (DeBruine, 2004). But men placed primary emphasis on cues of resemblance in a hypothetical adoption task (Volk and Quinsey, 2002) and in a task involved in making hypothetical parental investment decisions (Platek, 2002; Platek et al., 2003), while women mainly focused on cues of health and attractiveness (Volk and Quinsey, 2002). Therefore, self-other distinction associated regions such as the ventral medial prefrontal cortex (vmPFC) and dorsal medial prefrontal cortex (dmPFC) might be involved in the processing of infant faces (Mitchell et al., 2005; D’Argembeau et al., 2007).

Note that most of the functional imaging studies have focused on parental love, which limits the generalization of the findings to the non-parental care of infants. To further understand how gender influences the process of infant facial attractiveness, we conducted the present functional magnetic resonance imaging (fMRI) study investigating the neural mechanisms while males and females are viewing unknown infant faces. In the study, we manipulated the levels of infant facial attractiveness. To control general face-processing-related brain activations, we also included adult faces with average attractiveness. To reduce the confounding effects of age, marital status, as well as parenthood, only unmarried young female and male adults were recruited as participants. Based on previous findings, we expected to find different neural patterns while processing infant facial attractiveness in males and females, especially in the regions associated with reward processing, such as the striatum/NAcc and self-other distinction, such as vmPFC and dmPFC.

## Materials and Methods

### Participants

Thirty-two Chinese students (16 females, aged from 20 to 25, mean age = 22.8 ± 1.5 (SD) years; 16 males, aged between 19 and 26, mean age = 22.3 ± 1.8 years) from East China Normal University took part in the current study. All participants were unmarried. They were right-handed with normal or corrected-to-normal vision and had a similar level of education. They did not report any psychiatric or neurological history, and female participants were with regular menstrual cycles between 25 and 35 days. An 11-point scale (−5 = strongly dislike, 0 = neutral, 5 = strongly like) was used to assess the participants’ subjective feelings toward the infants before fMRI scanning. Female participants were arranged to take part in the experiment during the intervening period before and after the ovulation (i.e., 9 days before and 4 days after the ovulation). Female participants reported a mean of 3.1 days before the ovulation as calculated by their own menstrual phases. Written informed consents were obtained from all subjects, and the protocol was approved by the University Committee on Human Research Protection (UCHRP) at East China Normal University.

### Stimuli

One-hundred and eighty infant faces with neutral facial expression were selected from the Internet, an approach that has been used in previous studies (Brosch et al., 2007). The gender of the infant faces was not controlled as it was sometimes indistinguishable for babies (Proverbio et al., 2011). All pictures were transferred to gray-scale images with a black background of 640 × 480 resolution. The center between the two eyes was located at the same point to control for gaze (Nitschke et al., 2004). To make sure that the stimuli were suitable for testing our experimental hypotheses, all images were rated separately on a laptop by a separate group of 12 female participants for valence (with Self-Assessment Manikin, SAM), attractiveness (five-point scales ranging from 1 “not cute” to 5 “very cute”), gender (male/female) and age (five-point scales for infant faces ranging from 0 to 4 years old; five-point scales for adult faces ranging from 1 “16–20 years old” to 5 “36–40 years old”). The attractiveness of infant faces were almost equally distributed between 2.4 and 4.2 out of five points. The mean attractiveness of the female and male adult faces were 2.3 (SD = 0.5) and 2.0 (0.4), respectively. Thirty-six adult faces (18 females and 18 males) with neutral facial expression were also adopted as to examine whether the process involved in viewing the infant faces can be differentiated from the adult faces. There were more infant faces to enable the manipulation of the attractiveness of infant faces. Note that adult faces and infant faces were from different individuals since babies’ facial attractiveness cannot predict adult facial attractiveness of the same individuals (Harrison et al., 2011).

### Experimental Design

There were three functional runs. Each run lasted for 510 s and consisted of 10 blocks of infant faces and four blocks of adult faces (one for male and one for female, and each adult block was presented twice to increase signal-to-noise ratio). Each 18-s block consisted of six faces. Each face was presented for 3 s without inter-stimuli interval. During the 3-s of stimulus presentation, participants were asked to rate the attractiveness of the facial stimulus using a hand-shaped response box with their right hands, ranging from “1 = not attractive” to “5 = very attractive” with each rating was assigned to one finger. The inter-block fixation block lasted 18 s, with a fixation cross in the middle of the screen. The orders of these three kinds of block were pseudo-randomly mixed among three runs, and were counterbalanced between participants. This design was similar to Phan’s study (Phan et al., 2003). Stimuli were presented through a goggles system (Invivo Co., Gainesville, FL, USA).

### MRI Data Acquisition

The scanning was conducted on a 3-Tesla Siemens Trio MR scanner. For functional images, 35 axial slices (FOV = 240 × 240 mm^2^, matrix = 64 × 64, in-plane resolution = 3.75 × 3.75 mm^2^, thickness = 4 mm, without gap) covering the whole brain were obtained using a T2*-weighted echo planar imaging (EPI) sequence (TR = 3000 ms, TE = 30 ms, flip angle = 90°), with 170 volumes. A high-resolution structural image was also acquired for each participant using 3D MRI sequences for anatomical co-registration and normalization (TR = 1900 ms, TE = 3.43 ms, flip angle = 7°, matrix = 256 × 256, FOV = 240 × 240 mm^2^, slice thickness = 1 mm).

## Data Analysis

SPM8 was adopted for fMRI data analysis (Wellcome Department of Cognitive Neurology, London, UK)^1^. For each participant, the first two volumes of each run were discarded. EPI images were realigned to the first remaining volume of the first run to correct for head motions. Then the anatomical image was co-registered with the mean EPI image, segmented and then generated normalized parameters to MNI spaces. Next, all EPI data were projected to MNI template with a re-sampled voxel size of 2 × 2 × 2 mm^3^. Finally, the functional images were spatially smoothed with a Gaussian kernel with a full width at half maximum (FWHM) of 8 mm. High-pass temporal filtering with a cut-off of 128 s was carried out to remove low-frequency drifts.

The statistical analyses of the fMRI data were based on two General Linear Models (i.e., GLM 1 and GLM 2). The canonical hemodynamic response function was used to model the fMRI signal.

### GLM 1

The first GLM model (GLM 1) was set up to investigate gender differences in the neural processing of infant faces and adult faces. Two regressors of interest, i.e., adult faces and infant faces, were included. For adult faces, a boxcar model was used and adult attractiveness was not considered due to its small variety (SD = 0.5 and 0.4 for females and males respectively). For infant faces, as there are individual differences in perceiving facial attractiveness, an event-related parametric statistical model was used as an event-related analysis can provide a more accurate model of the hemodynamic responses than an epoch-related analysis, even in a blocked design (Büchel et al., 1998; Mechelli et al., 2003; Phan et al., 2003). An orthogonal basis functions up to second order were used (Büchel et al., 1998). The zero-order term modeled the main effect of infant faces to the crosshair regardless of the attractiveness. The first-order term modeled a parametric linear increase in participants’ subjective ratings of the attractiveness for each face, and a second-order term modeled a quadratic relationship. All these covariates were convolved with a canonical hemodynamic response function before including in the GLM model, and the six estimated head movement parameters were included in the design matrix to remove residual effects of head movements. The beta values of the adult face regressor, and the beta values of the zero-order term of the infant face regressors, were used as the interested indicators in the second level analysis.

For the second level analysis, a 2 × 2 flexible factorial model with the between-group factor (genders of the participants) and the within-subject factor (infant faces and adult faces) was built. Results were voxel-level height thresholded at *P* < 0.001 and survived after cluster-level family-wise error (FWE) correction, *P* < 0.05.

### GLM 2

A second GLM model (GLM 2) was set up to investigate the modulation of infant facial attractiveness on the neural activity in female and male participants. Infant faces were the regressor of interest. Participants’ ratings of attractiveness were entered as the parametric modulator. The six estimated head movement parameters were included in the design matrix to remove residual effects of head movements.

For the second level analysis, one sample *t*-tests were used to estimate the effects of the parametric modulator (i.e., infant facial attractiveness) respectively in female and male participants. Based on the findings of previous studies (Mitchell et al., 2005; D’Argembeau et al., 2007; Glocker et al., 2009b), our regions of interest are the striatum/NAcc, the vmPFC and the dmPFC. The voxel-wised threshold was set at *p* = 0.005, with the spatial extent threshold setting at *k* = 80. A small volume correction (SVC) for FWE was used in a box with dimensions equaling of 8 mm in brain regions with prior hypotheses. Specifically, the coordinates of the striatum/NAcc were 10 12 −8 (in Talairach space), adopted from Glocker et al’s (2009b) study, the coordinates of the vmPFC were −8 50 −2 (in MNI space; D’Argembeau et al., 2007), and the coordinates of the dmPFC were −9 51 36 (in MNI space; Mitchell et al., 2005). To investigate if the modulating effects differ between male and female participants, modulation coefficients were extracted from the aforementioned ROIs in males and females. Pair *t*-tests were used to compare modulation coefficients between males and females. The mean modulation coefficients of the vmPFC and striatum/NAcc for each participant were then extracted and used for correlation analysis, with the threshold setting at *P* < 0.05 (two-tailed).

## Results

### Behavioral Results

There was no significant gender difference in the ratings of liking for infants (women: 3.4 ± 1.5 (mean ± SD); men: 2.6 ± 1.7, *t*_(30)_ = −1.5, *p* = 0.14). Both groups gave significantly higher attractiveness ratings to infant faces than adult faces (females: 3.3 ± 0.6 vs. 2.4 ± 0.6, respectively, *t*_(15)_ = 5.3, *p* < 0.001; males: 3.1 ± 0.4 vs. 2.5 ± 0.5, respectively, *t*_(15)_ = 4.1, *p* = 0.001). We also found a significant correlation between subjective liking for infants and mean attractiveness of the infant faces in female participants (*r* = 0.75, *p* = 0.001) but not in male participants (*r* = 0.15, *p* = 0.58). No other effects were significant.

### Imaging Results

#### GLM 1

**Figure 1:**
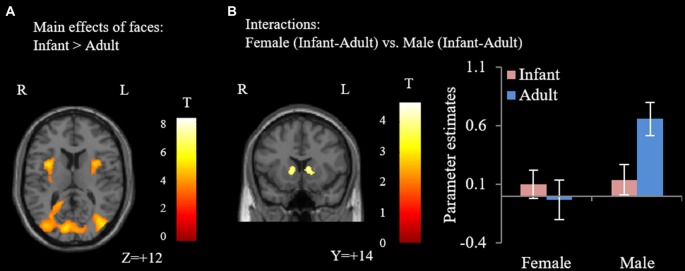
Results of the flexible factorial analysis. **(A)** Infant faces elicited higher activation in the bilateral insula and fusiform gyrus. **(B)** The interaction effect of female participants (infant-adult) vs. male participants (infant-adult). Error bars denote standard error of the mean (SE). Parameter estimates were extracted from the peak voxel in the striatum/nucleus accumbens (NAcc; MNI: 6, 2, 4; voxel-wised threshold *P* < 0.001, cluster-level *P* < 0.05, family-wise error (FWE) correction).

Our major findings were listed in the Table 1. When we compare the neural activity in female and male participants, no significant activations were found. With regards to the effects of faces, compared with adult faces, infant faces significantly activated bilateral insula and fusiform gyri (Figure 1A). Adult faces elicited higher activation in the bilateral middle frontal gyri, bilateral ventral lateral frontal gyri, bilateral superior parietal lobules, right middle temporal gyrus and bilateral precuneus, compared with infant faces. In the comparison of infants’ vs. adult faces, female participants showed higher activation in the striatum/NAcc (Figure 1B) compared with male participants. *Post hoc* analysis showed that adult faces elicited higher activity in male participants’ striatum/NAcc (*p*s ≤ 0.003). No significant differences were found in the other comparisons (*p*s > 0.14).

**Table 1:** Results of the flexible factorial analysis.

Hem	Volume^a^	Maxima location	MNI coordinates			*T*
**Main effects of gender**						
Female > Male						
None						
** Male > Female**						
None						
**Main effects of faces**						
Infant > Adult						
R	6502	Fusiform gyrus	34	−48	−6	8.24
R	616	Insula	32	6	14	5.35
L	308	Insula	−34	8	14	5.21
**Adult > Infant**						
R	8942	Middle frontal gyrus	36	22	32	7.01
R	809	Ventral lateral frontal gyrus	30	62	−2	6.95
L	2021	Superior parietal lobule	−42	−62	52	6.78
R	1954	Superior parietal lobule	36	−68	50	6.38
R	424	Middle temporal gyrus	56	−38	−12	6.38
L	1157	Ventral lateral frontal gyrus	−42	56	−4	5.90
M	1252	Precuneus	0	−58	24	5.53
**Interactions**						
Female (Infant-Adult) vs. Male (Infant-Adult)						
R	404	Striatum/NAcc	6	2	4	4.65
Male (Infant-Adult) vs. Female (Infant-Adult)						
None						

*^a^Volumes are given as number of 2.0 × 2.0 × 2.0 mm Voxels. Voxel-level height threshold P < 0.001, cluster-level P < 0.05, family-wise error (FWE) correction*.

#### GLM 2

**Figure 2:**
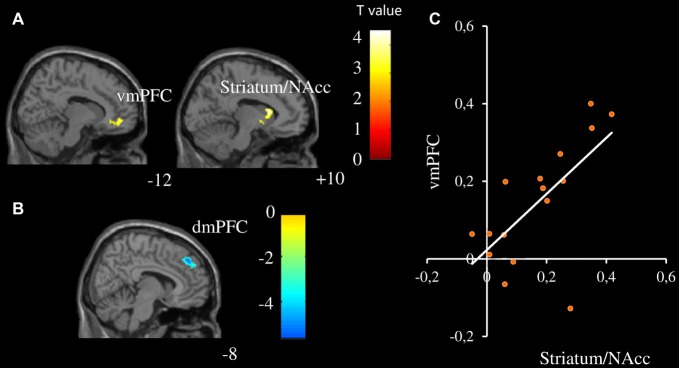
Neural activity was modulated by infant facial attractiveness differently between males and females. **(A)** The ventromedial prefrontal cortex (vmPFC) and striatum/NAcc were positively modulated by infant facial attractiveness in female participants, while **(B)** dorsomedial prefrontal cortex (dmPFC) was negatively modulated by infant facial attractiveness in male participants (*N* = 16, voxel-wised *p* < 0.005, uncorrected, *k* = 80; Small Volume Corrected). **(C)** A significant correlation between modulation coefficients of striatum/NAcc (centered at 10, 16, 10; with 808 ml) and vmPFC (centered at −12, 50, −8; with 832 ml) was found in female participants (*r* = 0.66, *p* = 0.006).

Different modulation patterns were found between female and male participants, see Table 2 for details. Specifically, the vmPFC and striatum/NAcc were positively modulated by infant facial attractiveness for female participants (Figure 2A), but not for male participants. Moreover, a positive correlation between the modulation coefficients of these two regions was found in female participants (Figure 2C). In male participants, the dmPFC was negatively modulated by infant facial attractiveness (Figure 2B), but not in female participants. The modulation coefficients in the vmPFC and striatum/NAcc were significantly higher in females than in males (also significant from these ROIs, *t*s_(30)_ > 1.7, *p*s < 0.05). In the dmPFC, the modulation coefficients were significantly higher in males than in females (*t*_(30)_ = 2.08, *p* = 0.023).

**Table 2:** Brain regions that were modulated by infant facial attractiveness.

Hem	Volume^a^	Maxima location	MNI coordinates			*T*
**Female (positive modulation)**						
R	101	Striatum/NAcc	10	16	10	4.52
L	104	vmPFC	−12	50	−8	3.96
**Male (negative modulation)**						
R	209	dmPFC	−8	44	40	5.53

*^a^Volumes are given as number of 2.0 × 2.0 × 2.0 mm voxels. Significant at p < 0.05 corrected for multiple comparisons at the cluster level with small volume correction (SVC)*.

## Discussion

In the present study, we found significant gender differences in neural responses toward infant and adult faces in two aspects. The first aspect is associated with the general processing of faces. While no gender differences were found when we compared neural activity in female and male participants, a significant gender (male and female) by face (infant vs. adult) interaction was found in the striatum/NAcc. Contrary to our expectations, *post hoc* analysis revealed significantly higher activity when male participants viewed adult faces. The striatum/NAcc is the key structure of the reward system, which can be activated by the monetary reward (Delgado et al., 2000; Elliott et al., 2003; Ernst et al., 2005; Abler et al., 2006; Knutson et al., 2001a,b, 2003) and social reward (Izuma et al., 2008; Spreckelmeyer et al., 2009). It is also associated with discrimination between reward values (Galvan et al., 2005), with higher reward (monetary reward or social reward) value eliciting higher striatum/NAcc activity (Izuma et al., 2008; Spreckelmeyer et al., 2009). According to these previous findings, there might be a strong link between reward and the activation in the striatum/NAcc. Thus, it seems that male participants displayed lower reward-related neural responses to infant faces (compared to adult faces). As a matter of fact, in a previous study, as cited in the Introduction, infant laughing and crying elicited activation in the amygdala and anterior cingulate of women, whereas the control stimuli elicited stronger activations in men (Sander et al., 2007). Their results, together with our results, suggest that males, but not females, display lower motivational neural responses to infant cues than to adult cues.

The second aspect is related to the modulation of attractiveness on the neural responses toward infant faces. The activities of the striatum/NAcc and vmPFC in female participants were positively modulated by infant facial attractiveness, while in male participants the activity of dmPFC was negatively modulated by infant facial attractiveness. With regards to infant face processing, our results on the striatum/NAcc are in line with the findings from a previous study in which it was found that cuter baby schema elicited higher NACC activation for female participants (Glocker et al., 2009b). Previous studies showed that, as compared to males, females were slightly better at detecting gradations in the manipulated attractiveness of infant faces (Sprengelmeyer et al., 2009; Lobmaier et al., 2010). Women gave significantly higher “liking” ratings for infant faces (Radin, 1982; Parsons et al., 2011) and display approach behavior toward infants (Frodi and Lamb, 1978). Furthermore, we found that the modulator coefficient in the vmPFC was correlated with that in the striatum/NAcc for female participants. The ventral striatum/NAcc (including NAcc) receives extensive projections from VMPFC (Delgado, 2007). Enhanced brain activation in the vmPFC was also found while seeing one’s own baby’s neutral face or hearing one’s own baby crying (Kringelbach et al., 2008; Noriuchi et al., 2008). The significant correlation between the vmPFC and striatum/NAcc suggests that these brain regions work together to initiate attractiveness induced likeness, especially in female participants. In females, the striatum/NAcc might be the key hub for initiating the attractiveness induced likeness.

A possible explanation is that self-other distinction is involved in the processing of infant facial attractiveness. Infant attractiveness was positively correlated with the activity of the vmPFC in female participants, but negatively correlated with the activity of the dmPFC in male participants. It was hypothesized that the vmPFC may be related to positive emotional evaluation. However, enhanced brain activation in the vmPFC was also found while seeing one’s own baby’s neutral face or hearing one’s own baby crying (Kringelbach et al., 2008; Noriuchi et al., 2008). In a study on self/other similarity (Mitchell et al., 2005), the vmPFC was activated during judgments about similar people, whereas the dmPFC was activated in judgments about dissimilar individuals. Similarly, activation of the vmDFC was found for judgments targeting the self (vs. judgments targeting the other), whereas the activation in the dmPFC was found when taking a third-person perspective compared to a first-person one (D’Argembeau et al., 2007).

The evidence about maternal love (Bartels and Zeki, 2004; Nitschke et al., 2004; Noriuchi et al., 2008; Swain, 2008) was consistent with the argument that the vmPFC was related to self-involvement in the processing of infants. Viewing attractive infant faces might induce higher involvement of “similar/self” system in female participants, while less involvement, or “disengagement”, of “dissimilar/other system” in male participants. Self might be the mediator of rewards (de Greck et al., 2008) in the processing of infant facial attractiveness. Through associating attractive babies with themselves, rather than others, women may feel more rewards toward cute infant faces. The explanation is in line with an evolutionary view that males have more evolutionary demands for this self-other distinction of babies because only males are susceptible to errors in identifying their offsprings (Platek et al., 2004). It has also been reported that young children were disproportionately at risk of homicide by step-parental males (Roach and Pease, 2011). Gender differences in caretaking behaviors show that women are more prone to physical care, either in families (Rossi, 1984; Baxter, 2002) or in social contexts other than family (Rossi, 1984). On the other hand, fathers act toward infants as if they are “things” rather than persons whom they can interact with; this is true even for egalitarian fathers (Rossi, 1984), in the sense that “others” are more likely to be treated as objects rather than human beings (Baars and Gage, 2010).

We also note that, behaviorally, participants of both genders considered infant faces to be more attractive than adult faces and we did not find significant differences between females’ and males’ ratings toward infant faces. Imaging results also showed that infant faces significantly activated bilateral insula and fusiform gyri (vs. adult faces, Figure 1A) in both genders without significant interactions. Taken together, these results suggest that participants’ ability to judge the attractiveness level of baby faces is comparable, while the striatum/NAcc and vmPFC/dmPFC motivational systems are differently involved in the processing of infant faces between males and females.

Our study bears several limitations. First, our study focused on unmarried adults. Therefore, it is unclear whether these differences are inherent or affected by culture. In addition, brain responses to infant facial attractiveness in other phases of the life cycle or in different marital/parental status (Proverbio et al., 2006) may differ. It is possible that reproductive hormones such as oxytocin may play a role in the evaluation of infant facial attractiveness (Sprengelmeyer et al., 2009), and parenthood may change the status of self-involvement as well as embodiment (Bault et al., 2011). Second, our sample size is relatively small (16 males and 16 females); further studies with larger sample sizes are desired. Third, the uneven number of trials corresponding to the infant and adult conditions respectively might influence statistical sensitivity. But we think that our findings are valid because our major conclusions are based on the findings from modulation analysis, in which adult faces were not included in the model.

In conclusion, we investigated the neural processing of infants’ and adults’ faces in females and males. Our major findings showed that: (1) females’ (but not males) likeness toward infants are highly associated with facial attractiveness; and (2) the neural processing of infant faces differ for two genders, especially in the regions of the striatum/NAcc, vmPFC, and dmPFC. The regions might be associated with attractiveness induced likeness and self-other distinction. Our findings thus provide hints for understanding established gender differences in baby processing.

## Author Contributions

LY, MF and ZW designed the study. LY and MF collected the data. LY and ZW analyzed the data. ZW, LY, LL and DS wrote the article.

## Conflict of Interest Statement

The authors declare that the research was conducted in the absence of any commercial or financial relationships that could be construed as a potential conflict of interest. The reviewer TT and handling Editor declared their shared affiliation, and the handling Editor states that the process nevertheless met the standards of a fair and objective review.

## Footnotes

^1^http://www.fil.ion.ucl.ac.uk/spm/
